# Indonesian stingless bee propolis extract attenuates hepatic inflammation following a chronic high-saturated fat diet

**DOI:** 10.1186/s12906-025-05236-8

**Published:** 2026-01-13

**Authors:** Andreas Christoper, Herry Herman, Rizky Abdulah, Felix Zulhendri, Milena Popova, Boryana Trusheva, Vassya Bankova, Ronny Lesmana

**Affiliations:** 1 Doctoral Program in Medical Science, Faculty of Medicine, Universitas Padjadjaran, West Java 40161 Bandung, Indonesia; 2 Department of Orthopaedics and Traumatology, Faculty of Medicine, Universitas Padjadjaran, West Java 40161 Bandung, Indonesia; 3 Universitas Padjadjaran Hospital, West Java 45360 Sumedang, Indonesia; 4 Department of Pharmacology and Clinical Pharmacy, Faculty of Pharmacy, Universitas Padjadjaran, West Java 45363 Sumedang, Indonesia; 5 Center of Excellence in Higher Education for Pharmaceutical Care Innovation, Universitas Padjadjaran, West Java 45363 Sumedang, Indonesia; 6 Kebun Efi, North Sumatra 22171 Kabanjahe, Indonesia; 7 Institute of Organic Chemistry with Centre of Phytochemistry, Bulgarian Academy of Sciences, Sofia, 1113 Bulgaria; 8 Physiology Division, Department of Biomedical Sciences, Faculty of Medicine, Universitas Padjadjaran, West Java 40161 Bandung, Indonesia; 9 Division of Biological Activity, Central Laboratory, Universitas Padjadjaran, West Java 45363 Sumedang, Indonesia

**Keywords:** Dyslipidemia, Hepatic inflammation, High-fat diet, Hyperglycemia, Indonesian propolis extract

## Abstract

**Background:**

Metabolic syndromes, including obesity, dyslipidemia, and diabetes mellitus, can exacerbate inflammation-induced liver damage, which is associated with high-fat diets (HFD) and sedentary lifestyles. The substantial hypolipidemic and anti-inflammatory properties of propolis extract can potentially reduce hepatic inflammation and prevent the progression of liver damage. This study aims to investigate the role of Indonesian propolis extract in reducing hepatic inflammation after dyslipidemia and hyperglycemia.

**Methods:**

The experiment started with feeding HFD to the rats for 12 weeks, then a daily dosage of 300 mg/kg BW of propolis extract was administered via gavage for 9 weeks. Body weight, liver index, and blood biochemical analysis were conducted to determine the pharmacodynamic evaluation of propolis extract. The expression of inflammatory cytokines was investigated in the liver tissue. The correlation and regression analysis assessed the relationship between dyslipidemia, hyperglycemia, and hepatic inflammation.

**Results:**

Nine weeks of propolis extract supplementation significantly decreased body weight and liver index. Propolis also improves blood profile by reducing cholesterol, triglycerides, LDL-C, and glucose levels and increasing HDL-C levels. The expression of hepatic inflammatory cytokines such as IL-1β, IL-6, and TNF-α was decreased in the HFD group after propolis administration. A significant moderate to strong correlation (0.56–0.94) was found between the lipid profile, glucose levels, and hepatic inflammation. The lipid profile represented approximately 84.6%, 49.9%, and 68.7% of the hepatic inflammatory cytokine expression variation of IL-1β, IL-6, and TNF-α.

**Conclusion:**

Based on current evidence, Indonesian propolis alleviated hepatic inflammation by improving dyslipidemia and hyperglycemia after a prolonged high-fat diet in vivo. Therefore, further clinical studies are warranted to substantiate its complementary role in metabolic or inflammatory conditions.

**Clinical trial number:**

Not Applicable.

## Introduction

Non-communicable diseases have become a concern around the world due to their associated morbidity, mortality, and disability. Metabolic syndrome, including obesity, dyslipidemia, and type 2 diabetes mellitus, is a risk factor for non-communicable illnesses. The World Health Organization asserts that millions of deaths, disabilities, and cases of dependency can occur annually in the world due to imbalances in the plasma lipid profile and fasting blood glucose. The increasing prevalence of these metabolic syndromes worldwide has been strongly associated with dietary changes, notably the intake of high-fat diets (HFD). Frequent and excessive consumption of foods high in saturated and trans-fats and sedentary lifestyles can promote organ damage due to dyslipidemia and hyperglycemia via several processes and pathways related to inflammation [1, 2]. 

Dyslipidemia is characterized by an imbalance of lipid profiles, such as high levels of total cholesterol, triglycerides, low-density lipoprotein cholesterol (LDL-C), and low levels of high-density lipoprotein cholesterol (HDL-C). An unhealthy lifestyle, such as high consumption of high-calorie diets and low physical activity, results in energy storage in lipids in white adipose tissue [3]. Chronic high-fat food intake can cause ectopic organs like the liver to substitute adipose tissue and skeletal muscle as the principal energy storage source. The process includes high transport of free fatty acids (FFAs) from chylomicrons to adipose tissue, the conversion of triacylglycerol from adipose tissue to diacylglycerols in hepatocytes via fatty acid transport protein. The FFAs converted from lipids are then stored in hepatocytes as triglycerides. Excessive accumulation of triglycerides can cause lipotoxicity, eventually generating liver injury by promoting oxidative stress and inflammation [4]. 

Lipotoxicity can generate reactive oxygen species (ROS) by increasing the β-oxidation of FFAs in hepatic mitochondria, resulting in oxidative stress. Elevated ROS can trigger inflammasomes, leading to hepatocyte mitochondrial damage and inflammation [5]. The inflammation of hepatocytes involves the endoplasmic reticulum stress due to abundant FFAs and Kupffer cells activation, leading to the activation of nuclear factor kappa B (NF-κB) and c-Jun N-terminal kinase (JNK) pathway to increase the production of pro-inflammatory cytokines such as Interleukin-1 beta (IL-1β), Interleukin-6 (IL-6), Tumor Necrosis Factor-alpha (TNF-α), and Transforming Growth Factor-beta (TGF-β) in the liver [6]. The activated pathway modulates not only inflammation but also insulin resistance, both in the liver and circulation.

Hyperglycemia is a metabolic syndrome related to insulin resistance, aggravated by continuous HFD intake. HFD-induced insulin resistance inhibits glucose absorption in the peripheral tissues, such as adipose tissue and skeletal muscle, leading to hepatic gluconeogenesis and high fasting blood glucose levels [7]. Interestingly, this condition activates *de novo* lipogenesis (DNL), which can cause high production of FFAs, ROS, and inflammatory cytokines. *De novo* lipogenesis later aggravates lipotoxicity and worsens hepatic inflammation [8]. Chronic hyperglycemia also elevates the synthesis of advanced glycation end-products, contributing to liver tissue inflammation and oxidative stress [9]. Furthermore, the relationship involving a high-fat diet, dyslipidemia, hyperglycemia, and liver inflammation emphasizes the necessity of regulating nutrition and metabolic health to avert the advancement of non-alcoholic fatty liver disease (NAFLD) and other metabolic disorders. Hepatic inflammation and NAFLD are related to the ‘multiple hit’ concepts, which include HFD, dyslipidemia, and hyperglycemia. The interaction between insulin resistance-related hyperglycemia and dyslipidemia to liver inflammation includes the lipotoxicity related to high FFAs and *de novo *lipogenesis activation, mitochondrial dysfunction, oxidative stress, and activation of the inflammasome and proinflammatory cytokines. These pathways lead to liver inflammation, steatohepatitis, and NAFLD, which later induce hepatocyte damage, apoptosis, and fibrosis [10, 11]. Comprehending these processes is essential for pinpointing prospective treatment targets and formulating therapies to mitigate liver-related mortality and morbidity.

Nowadays, apitherapy has been widely used in complementary treatment to control some progressive diseases. Apitherapy uses bee products, such as bee honey, royal jelly, and propolis, which contain antioxidant or anti-inflammatory properties that might support the hepatocytes in eliminating inflammation in dyslipidemia. Propolis is one of those potential products with the highest antioxidant and anti-inflammatory properties among all bee products. These properties are advantageous in treating and preventing inflammation-related diseases [12, 13]. In this case, addressing dyslipidemia and hyperglycemia is beneficial in reducing liver inflammation.

Certain research has demonstrated that propolis extract helps address dyslipidemia and hyperglycemia. Turkey propolis treatment was found to have an effect in lowering total cholesterol, triglycerides, and blood glucose in ApoE^−/−^ mice after a high-cholesterol diet [14]. In mice fed a high-fat diet, an ethanolic extract of Croatian propolis at a dosage of 50 mg/kg BW reduced several lipid markers, including total cholesterol, triglycerides, and LDL-C, as well as glucose levels in blood and liver tissue [15]. Moroccan propolis at doses of 50 and 100 mg/kg BW significantly reduced blood glucose levels and modified some lipid profile markers in diabetic rats [16]. Another study utilizing Nigerian propolis found that there is a significant increase in HDL-C levels and a decrease in glucose and VLDL levels following 5-week propolis treatment at doses of 300 mg/kg BW in an alloxan-induced diabetic rat model [17]. Therefore, we propose that propolis extract might reduce hepatic inflammation by modulating the lipid profile and blood glucose level.

Our research employed propolis extract derived from *Geniotrigona thoracica* stingless bees in North Sumatra, Indonesia, since we posited that this extract might mitigate hepatic inflammation, supported by our prior investigations. We found that Indonesian glyceric extract of propolis (GEP) decreased pathogenic bacterial growth, improved gut microbiota homeostasis, and repaired intestinal structure after chronic HFD exposure, which might be related to inflammatory treatment [18]. We also discovered that the propolis extract reduced neuroinflammation after chronic high-fat diet intake by reducing body weight and restoring autophagy flux [19]. The impact of Indonesian GEP’s antioxidant and anti-inflammatory properties on liver inflammation linked to dyslipidemia and hyperglycemia has not been thoroughly studied. Therefore, we are interested in assessing how well it can reduce inflammation following these conditions, especially in the liver.

## Materials and methods

### Animal studies

This experimental study was approved with registration number 822/UN6.KEP/EC/2022 by the Research Ethics Committee (Universitas Padjadjaran, Bandung, Indonesia). Eight-week-old Wistar rats (*n* = 20) were supplied from Biofarma Company, Bandung, Indonesia. The rats were kept and maintained as research subjects at the Animal Science Laboratory, Graduate School, Universitas Padjadjaran. The cage was controlled in suitable conditions, including humidity (40–70%), temperature (24–26 °C), and 12-hour dark-light cycles. The rats were acclimatized for 2 weeks before the experiment.

The rats were equally divided into the control/normal chow diet group (CD), control with propolis treatment (CP), high-fat diet (FD), and high-fat diet with propolis treatment (FP). The normal-chow and high-fat diet pellets (Prospets^®^) were obtained from Surya Sains Indonesia Company, Bandung, Indonesia. The standard calorie intake and diet composition are described in Table 1. The rat body weight measurement was conducted weekly. The experiment was performed with a 21-week high-fat diet (HFD) in the FD and FP groups, with the last 9 weeks of glyceric extract of propolis (GEP) treatment for each rat in the CP and FP groups. The propolis was administered via intragastric gavage daily at a dosage of 300 mg/kg BW. The rats were euthanized and sacrificed after the experiment, using isoflurane 5% inhalation euthanasia methods. The liver index was calculated by weighing the liver and dividing it by the body weight. The liver organ was then kept at − 80 °C in the sealed tubes and labeled according to the groups. Some liver portions were extracted for Multiplex ELISA examination.

**Table 1:**
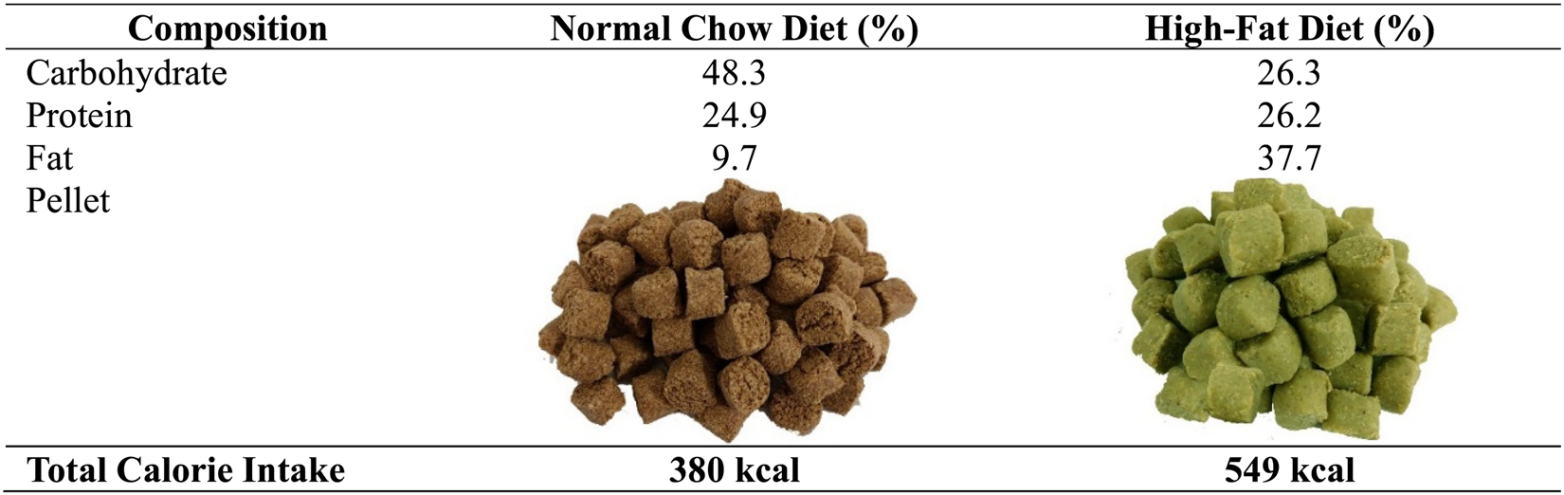
Calorie intake and food composition of the rat diet

### Propolis extract preparation and administration

The hydroglyceric stingless bee (*Geniotrigona thoracica*) propolis extract was supplied by Efi Maju Sejahtera Company, North Sumatra, Indonesia. The propolis extract was prepared using a proprietary method that involves multiple heating and filtration steps of the raw propolis collected from the hives, where the end product contains 15.5% w/v (propolis: hydroglycerol). Then, we analyze the components of the glyceric extract of propolis using Gas Chromatography-Mass Spectrometry (GC-MS), with similar methods described in a prior publication [19]. The dose of propolis given to each rat in the CP and FP groups was 300 mg/kg BW. The rats received daily intragastric propolis gavage. The dose of propolis was evaluated weekly based on the rats’ body weight.

### Blood biochemical analysis

The blood for the lipid profile and ad-random glucose levels analysis was drawn from the tail after 12 weeks of a high-fat diet (approximately 0.5 ml) and from the heart at the end of the experiment (approximately 1.5 ml). The collected blood was centrifuged at 10,000 × *g* for five minutes at 4 °C to extract the serum. The lipid profile (total cholesterol, triglycerides, LDL-C, HDL-C) and glucose levels were analyzed from the serum using a commercial kit (DiaSys Diagnostic Systems GmbH, Holzheim, Germany). The experimental procedure followed the manufacturer’s guidelines.

### Inflammatory cytokines analysis

The liver tissues (± 10 mg) from all groups (*n* = 20) were homogenized in a Sodium Dodecyl Sulfate (SDS) Lysis Buffer Solution. The supernatants were collected and analyzed after centrifugation of liver homogenate (10,000 × *g*, 4 °C, 10 min). The MILLIPLEX MAP Rat Cytokine Magnetic Bead Immunoassay (RECTYMAG-65 K-03, Millipore, Billerica, MA, USA) was used to analyze the levels of IL-1β, IL-6, and TNF-α from liver protein supernatants. The experimental procedure followed the manufacturer’s guidelines. The Median Fluorescent Intensity data were acquired using the Luminex^®^ MAGPIX^®^ instrument with xPONENT^®^ software.

### Statistical analysis

The data were processed in SPSS Statistics V24 and demonstrated with mean ± standard error of the mean. Following 12 weeks of HFD intake, the lipid profiles of the CD and FD groups were compared using an independent sample *t*-test analysis to determine if there were any notable changes. To identify the statistical differences and significance of the results between the CD, CP, FD, and FP groups, a one-way ANOVA with Tukey’s post hoc test was implemented. The causal association between inflammation and dyslipidemia was discovered using Pearson’s correlation. The effect of the lipid profile on hepatic inflammation was estimated using multiple linear regression analysis. On the other hand, the impact of glucose levels on hepatic inflammation was evaluated using simple linear regression analysis. The data were considered significant if the *p*-value was less than 0.05.

## Results

### Indonesian propolis extract from North Sumatra mostly contained triterpenoids

The result of GC-MS showed that the propolis extract mainly consists of triterpenes, such as cycloartenol, α-amyrenone, and α-amyrine. The composition of the propolis extract is described in Table 2.

**Table 2:** The composition of Indonesian hydroglyceric stingless bee propolis extract

Retention Time	Compound^1^	% of TIC^2^	M^+^	Prominent MS Peaks ^3^, m/z
	***Cardanols***			
32,93	Cardanol 17:2 (C_17_H_31_)	0.2	400	180 (100)
33,27	Cardanol 17:0 (C_17_H_35_)	0.2	404	180 (100)
36,03	Cardanol 19:1 (C_19_H_37_)	0.6	430	180 (100)
	***Cardols***			
33,70	Cardol 15:0 (C_15_H_31_)	0.5	464	268 (100)
35,88	Cardol 17:2 (C_17_H_31_)	1.2	488	268 (100)
36,03	Cardol 17:1 (C_17_H_33_)	0.6	490	268 (100)
36,33	Cardol 17:1 (C_17_H_33_) isomer	1.1	490	268 (100)
36,24	Cardol 17:0 (C_17_H_35_)	0.7	492	268 (100)
	***Anacardic acids***			
38,11	Anacardic acid C 17:1 (C_17_H_35_)	0.1	518	503 (100), 219
38,17	Anacardic acid C 17:1 (C_17_H_35_) isomer	0.2	518	503 (100), 219
38,30	Anacardic acid C 17:0 (C_17_H_35_)	0.8	520	505 (100), 219
	***Triterpenes***			
42,23	β-Amyrenone	2.4	424	218 (100), 203, 189
42,66	β-Amyrine	2.9	498	218 (100), 203, 189
42,96	*α*-Amyrenone	7.0	424	218 (100), 203, 189
43,23	*α*-Amyrine	3.6	498	218 (100), 203, 189
43,43	Cycloartenol	10.2	498	408 (100), 393, 365, 339
55,63	Mangiferolic acid	2.6	600	585, 510, 495, 467 (100), 441, 388
	***Aromatic aldehydes***,*** aromatic acids***,*** and their esters***			
15,04	Glyceryl caproate	0.2	Not visible	231, 147, 99 (100)
18,32	Glyceryl caprylate	0.2	Not visible	259, 147, 127 (100)

^1^ The name given does not include TMS substituents; ^2^ The ion current generated depends on the characteristics of the compound concerned and is not an accurate quantification; ^3^ (100)–base peak

### The lipid profile was significantly altered after propolis administration

**Fig. 1:**
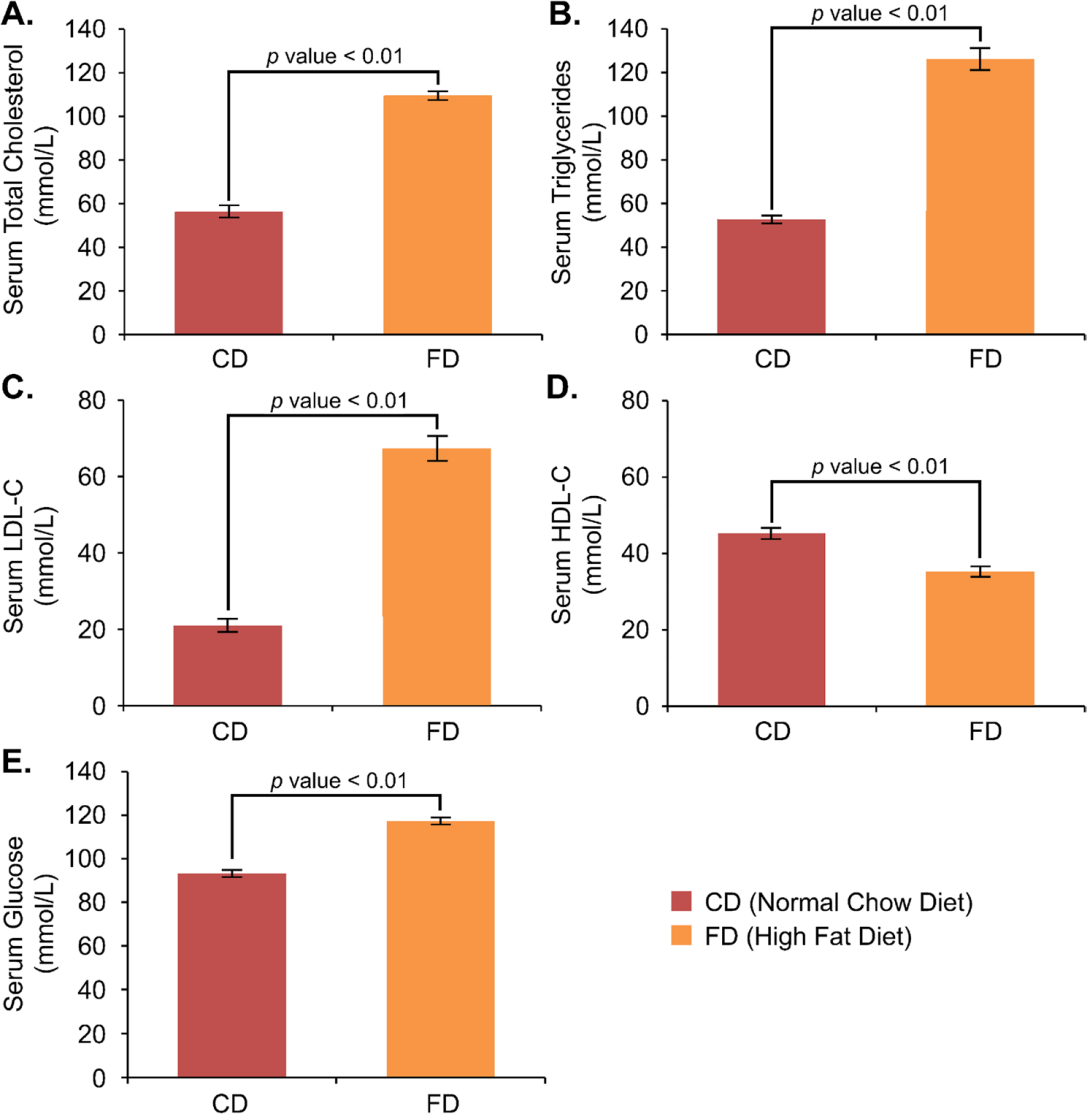
The serum examination results after 12 weeks of High-Fat Diet (HFD). The lipid profile and glucose level were analyzed in all groups, such as (**A**) total cholesterol, (**B**) triglycerides, (**C**) LDL-C, (**D**) HDL-C, and (**E**) glucose levels. Data are presented as mean ± standard error of the mean, with a significance level of < 0.01 between groups

We examined the lipid profiles and serum glucose of all groups after 12 weeks of HFD and 9 weeks of GEP treatment. The serum total cholesterol (TC), triglycerides (TG), and LDL-C levels were significantly increased, and the HDL-C levels were significantly decreased in the high-fat diet group compared to the control group. Figure 1 describes the lipid profile results after a 12-week high-fat diet. TC and TG levels of the high-fat diet group were higher (TC: 109.37 mmol/L; TG: 126.27 mmol/L) than those of the control group (TC: 56.19 mmol/L; TG: 52.81 mmol/L). The LDL-C levels (67.36 mmol/L) were higher, but the HDL-C levels (35.22 mmol/L) were lower in the high-fat diet group than in the control group (LDL-C: 21.07 mmol/L; HDL-C: 45.23 mmol/L). The lipid profile was significantly changed after a 12-week high-fat diet (*p* < 0.01).

**Fig. 2:**
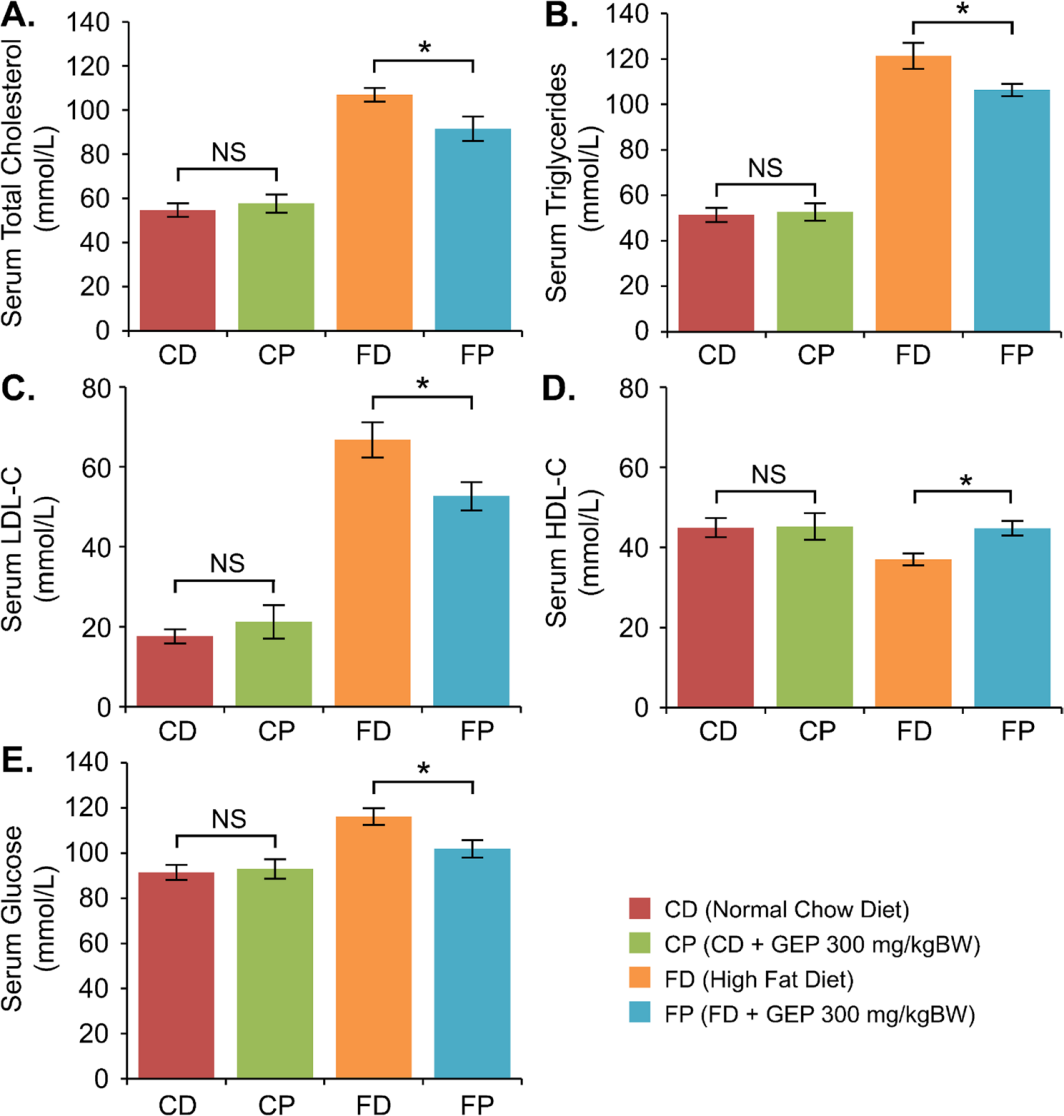
The serum examination results after 21 weeks of the experiment. The lipid profile and glucose level and were analyzed in all groups, such as (**A**) total cholesterol, (**B**) triglycerides, (**C**) LDL-C, (**D**) HDL-C, (**E**) glucose levels. Data are presented as mean ± standard error of the mean, *p < 0.05 vs. FD group, NS: not significant

After 9 weeks of GEP treatment, the lipid profile changed significantly in the FP group compared to the FD group. In our study, GEP significantly altered the serum lipid profile in the FP group compared to the FD group after nine weeks of treatment (Fig. 2), such as TC (91.54 vs. 106.94 mmol/L), TG (106.40 vs. 121.42 mmol/L), LDL-C (52.68 vs. 66.78 mmol/L), and HDL-C (44.75 vs. 36.92 mmol/L). However, even after 21 weeks of the experiment, the lipid profile levels of the FP group, except for HDL-C, were greater than those of the control (CD and CP) group. The lipid profile levels of the CD and CP groups were as follows: TC: 54.67 and 57.71 mmol/L; TG: 51.47 and 52.68 mmol/L; LDL-C: 17.64 and 21.26 mmol/L; and HDL-C: 44.90 and 45.26 mmol/L.

Furthermore, we discovered that a high-fat diet might elevate glucose levels (GLU) besides altering lipid profiles. As presented in Fig. 1E, serum glucose levels differed significantly between the high-fat diet group (117.35 mmol/L) and the normal chow diet group (93.19 mmol/L). Nine-week GEP treatment significantly altered the glucose level after chronic high-fat diet consumption, demonstrated by lower glucose levels in the FP group (101.97 mmol/L) than in the FD group (116.19 mmol/L). However, glucose levels in the FP group were still higher than in the CD (91.42 mmol/L) and CP (92.97 mmol/L) groups.

### The body weight, liver index, and hepatic inflammatory cytokine expression were reduced after propolis administration

**Fig. 3:**
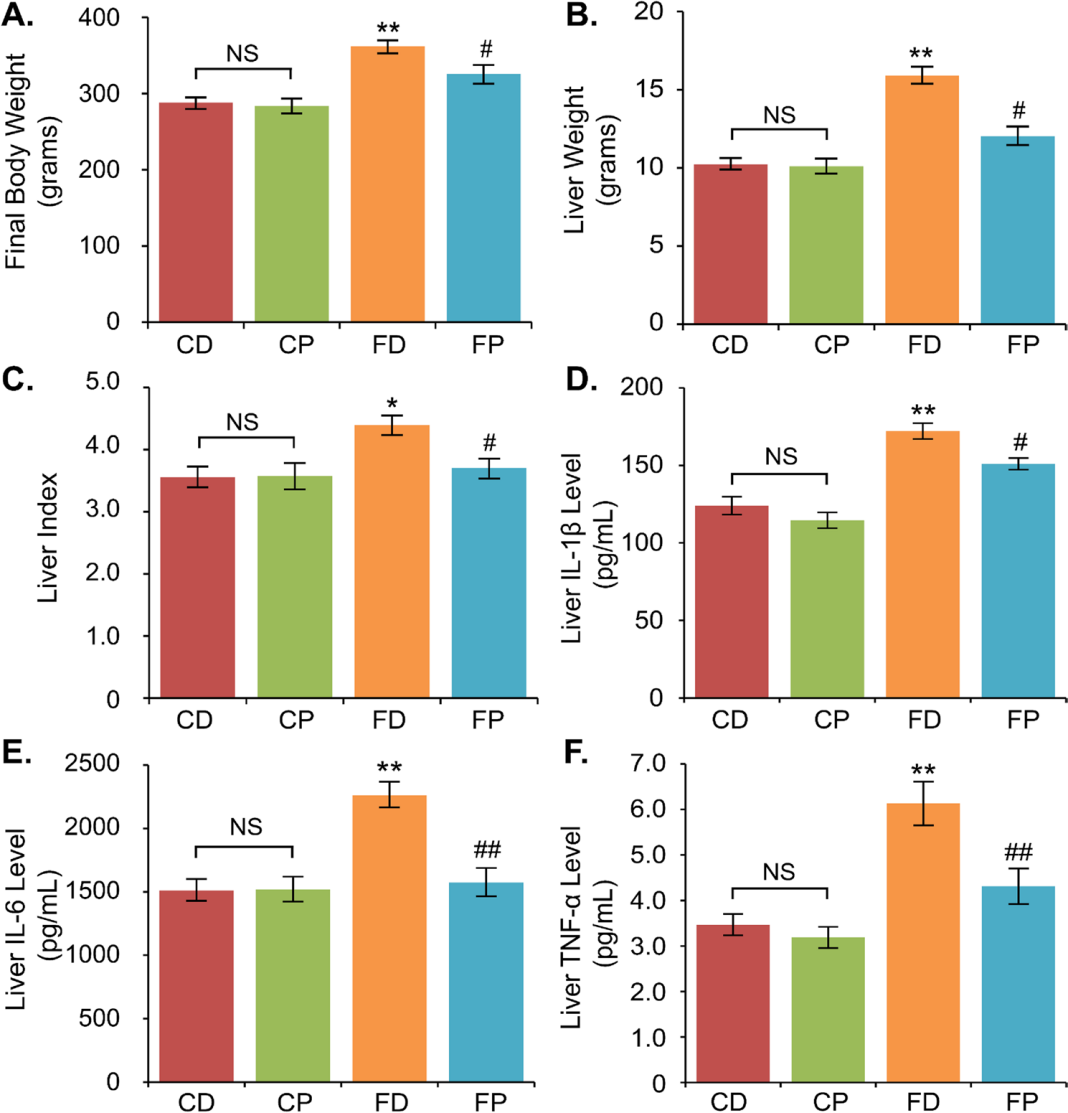
GEP altered body weight, liver index, and inflammation biomarkers. The final mean body weight (**A**), liver weight (**B**), and liver index (**C**) were measured after 21 weeks of the experiment. The inflammatory cytokines such as IL-1β (**D**), IL-6 (**E**), and TNF-α (**F**) were analyzed from the liver tissues in all groups. Data are presented as mean ± standard error of the mean, *p < 0.05 and **p < 0.01 vs. the CD group, and ^#^*p* < 0.05 and ^##^*p* < 0.01 vs. the FD group, NS: not significant

We discovered that HFD significantly increased body weight (BW) in the FD group (361.8 g) compared to the control group (CD: 288 g; CP: 283.6 g) after 21 weeks of the experiment (Fig. 3). Liver weight (LW) and liver index (LI) were also higher in the FD group (LW: 15.89 g, LI: 4.4) than in CD (LW: 10.22 g, LI: 3.56) and CP (LW: 10.1 g, LI: 3.58) groups. 9-week propolis administration decreased body weight, liver weight, and liver index after chronic HFD, as seen in the FP group (BW: 325.8 g; LW: 12.02 g; LI: 3.70).

We then analyzed the inflammatory cytokine expression in the liver tissues, such as IL-1β, IL-6, and TNF-α, using multiplex ELISA after 21 weeks of the experiment, including 12 weeks of a high-fat diet and 9 weeks of GEP treatment. High-fat diet promoted inflammation in the liver, proven by higher inflammatory cytokines expression such as IL-1β levels (172.18 pg/mL), IL-6 levels (2262.07 pg/mL), and TNF-α (6.13 pg/mL) levels compared to the control (CD and CP) groups (IL-1β: 124 and 114.55 pg/mL; IL-6: 1510.81 and 1517.90 pg/mL, and TNF-α: 3.47 and 3.19 pg/mL). Nine-week treatment of GEP in FP groups significantly reduced the expression of the inflammatory cytokines after 12-week HFD consumption, such as IL-1β (150.96 pg/mL), IL-6 (1572.82 pg/mL), and TNF-α (4.31 pg/mL).

### There is a relationship between dyslipidemia, hyperglycemia, and liver inflammation

**Fig. 4:**
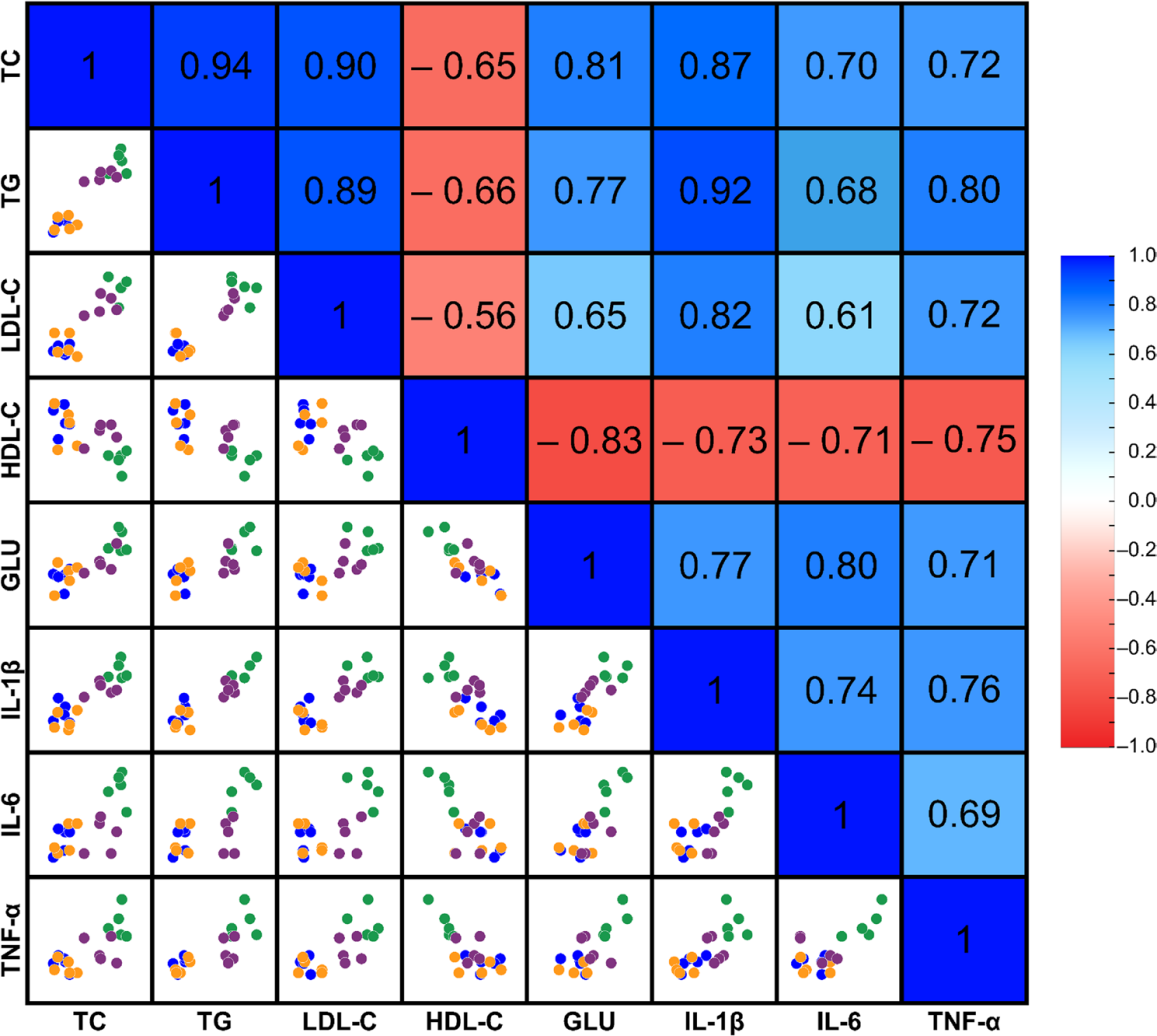
Pearson’s correlation analysis heatmap and matrix scatter/dot chart between dyslipidemia, hyperglycemia, and hepatic inflammation. The different colour dots represent the study group, such as blue (CD), orange (CP), green (FD), and purple (FP)

The Pearson’s correlation analysis identified the relationship between lipid profile, glucose level, and inflammation in the liver. In this analysis, we examined the lipid profile after 21 weeks of experiment and inflammatory cytokine expression of the liver. The lipid profile measures, including TC, TG, LDL-C, HDL-C, and glucose (GLU) levels, significantly correlated with the production of liver inflammatory cytokines, such as IL-1β, IL-6, and TNF-α (Fig. 4).

**Fig. 5:**
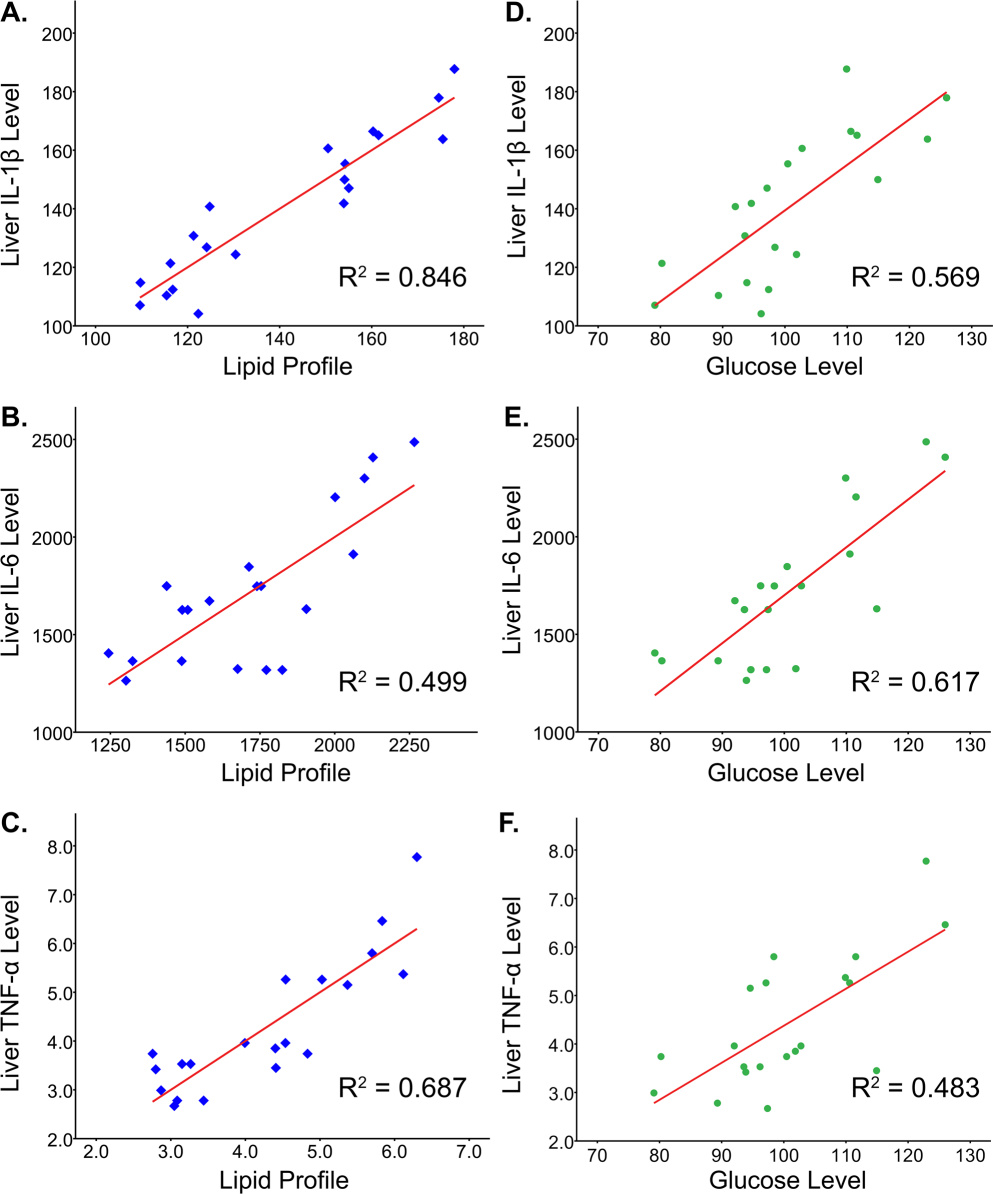
The regression analysis explores the relationship between lipid profile, glucose level, and inflammatory cytokines. The multiple linear regression analysis was conducted to examine the effect of lipid profile on hepatic inflammatory cytokines expressions such as IL-1β (**A**), IL-6 (**B**), and TNF-α (**C**). In contrast, simple linear regression analysis assessed the effect of glucose levels on hepatic inflammatory cytokines expressions such as IL-1β (**D**), IL-6 (**E**), and TNF-α (**F**)

Multiple linear regression analysis was used to test whether the lipid profile significantly predicted the expression of IL-1β, IL-6, and TNF-α in the liver. We observed that the lipid profile represented approximately 84.6%, 49.9%, and 68.7% of the hepatic inflammatory cytokine expression variation of IL-1β, IL-6, and TNF-α. Meanwhile, simple linear regression analysis estimated the relationship between glucose level and hepatic inflammation and found that the glucose level accounted for nearly 56.9% (IL-1β), 61.7% (IL-6), and 48.3% (TNF-α) of the variation in hepatic inflammation (Fig. 5).

## Discussion

A high-fat diet (HFD), especially high in saturated fatty acids, harms human health because it can induce several metabolic disturbances, such as dyslipidemia and hyperglycemia. Chronic HFD and sedentary lifestyles increase the risk of organ damage and failure. One of the organs impacted by a chronic HFD is the liver, which is essential in lipid metabolism. High circulating free fatty acids (FFAs) and insulin resistance are the primary keys to liver inflammation and damage. We used HFD in the experiment for 12 weeks to induce dyslipidemia and hyperglycemia in the rats. As it should be, the lipid profile and glucose level were significantly altered in the FD group compared to the control group. Except for HDL-C, serum TC, TG, LDL-C, and glucose levels were higher after 12 weeks of HFD consumption. The discovery was similar to some animal studies that showed an increased lipid profile after 6 weeks of HFD [20, 21].

This study revealed that in addition to dyslipidemia and hyperglycemia, a 21-week HFD resulted in increased body weight, liver weight, and hepatic inflammation. The level of hepatic inflammatory cytokines significantly increased after a chronic high-fat diet, similar to other studies that found inflammation due to obesity and dyslipidemia after induction of a high-fat diet. Yan et al. discovered high expression of inflammatory cytokines in serum and liver after 16weeks of a high-fat diet. They also found that HFD provoked inflammation via inflammasomes and central inflammatory pathways, such as NLRP3/ASC/caspases and TLR4/Myd88/NF-κB [22]. Yuan et al. (2020) found an increase in IL-6 and TNF-α after 14–18 weeks of HFD, which is related to pathological liver histology [23]. These studies also found a significant alteration of serum lipid profile and glucose in the HFD group compared to the control group. Thus, we expected an association between inflammatory events in the liver, dyslipidemia, and hyperglycemia.

This study aims to prove the protective effect of Indonesian glyceric extract of propolis on the liver after a chronic high-fat diet. This study used a glyceric extract of propolis (GEP) from *Geniotrigona thoracica* in North Sumatra, Indonesia, which was rich in triterpenes, such as cycloartenol, amyrenone, amyrine, and mangiferolic acid. The composition of propolis obtained from tropical countries, such as Indonesia, Myanmar, Malaysia, and other Southeast Asian countries, mainly consists of triterpenes. Although abundant studies mention the benefits of phenolic acid and flavonoids in organ health, terpenoids also have several advantageous properties, such as antibacterial, antioxidant, and anti-inflammatory, where there are only a few studies investigating the role of terpenoid-rich propolis [24, 25]. To our knowledge, this is the first study to discover the effect of high-terpenoid propolis on HFD-induced metabolic and hepatic disorders.

Interestingly, we found that a 9-week treatment with glyceric extract of propolis (GEP) could improve the lipid profile in the FP group, although it was still higher than that in the control group. The treatment of GEP improved dyslipidemia and hyperglycemia, as indicated by a substantial reduction in total cholesterol, triglycerides, LDL-C, glucose levels, and an elevation in HDL-C levels in the FP group relative to the FD group. Concurrent with this study, some studies also stated that propolis could improve dyslipidemia and hyperglycemia in chronic HFD and other metabolic diseases [15, 26]. The efficacy of propolis in metabolism warrants further exploration since chronic metabolic disorders may result in organ degeneration. Moreover, our findings show that this is the first article to find that the glyceric extract of propolis high in terpenoid content can ameliorate hepatic inflammation after chronic saturated fat exposure from HFD. Some articles supported this study, mentioning that propolis could effectively decrease HFD-induced either hepatic or systemic inflammation [27, 28]. The study demonstrated that Indonesian propolis alleviated liver inflammation by improving lipid profiles after a chronic high-fat diet.

Furthermore, we found that there was a relationship between dyslipidemia, hyperglycemia, and liver inflammation. There were moderate to high correlations between serum lipid profile, serum glucose level, and liver inflammatory cytokine levels. We also examined using regression analysis that lipid profile accounted for 84.6%, 49.9%, and 68.7%, and glucose level for 56.9%, 61.7%, and 48.3% variability in hepatic IL-1β, IL-6, and TNF-α expression. Our findings validate the ‘multiple-hit’ hypothesis, suggesting that dyslipidemia and hyperglycemia contribute to liver inflammation progression, which might relate to steatohepatitis and NAFLD [10, 29, 30]. Research has widely utilized a high-fat diet to determine the impact of FFAs on liver damage. The FFAs can damage the liver by promoting inflammation and oxidative stress, with the damage occurred concurrently with elevated circulating cholesterol and triglycerides [14, 31]. 

We postulated the mechanism from what we found in the study, supported by other findings from the literature. A long-term high-fat diet can cause increased chylomicron (CM) production and disrupt the microbiota in the intestine. It will induce intestinal dysbiosis and produce lipopolysaccharide (LPS), which will then circulate in the bloodstream [32, 33]. Chylomicrons in the circulation are broken down into FFAs by lipoprotein lipase. Circulating LPS and FFAs bind to TLR-4 macrophages and cause M0 polarization to M1, which actively releases proinflammatory cytokines, leading to systemic inflammation [34]. These remaining chylomicrons (CMr) are transported and broken down in the liver in the form of cholesterol [35]. The circulating FFAs then enter various peripheral organs, such as skeletal muscle and adipose tissue, to store energy in triglyceride form via lipogenesis. Analogous to circulation, FFAs in adipose tissue induce the polarization of adipose tissue macrophages (ATMs) into M1 macrophages, resulting in heightened production of proinflammatory cytokines that contribute to chronic inflammation. However, excess delivery and accumulation of FFAs in adipose tissue prompt the lipolysis of triglycerides, resulting in the formation of FFAs and glycerol for transport to the liver [36–38]. 

On the other hand, circulating glucose obtained from the diet should enter peripheral tissues such as skeletal muscle to be used as an energy source. Elevated concentrations of FFAs infiltrating skeletal muscle will disrupt insulin-mediated glucose transport, inhibiting glucose uptake by cells and resulting in hyperglycemia. High glucose levels in the bloodstream will increase insulin production, leading to insulin resistance. Insulin resistance and high glucose levels will trigger *de novo lipogenesis* (DNL) in the liver, which will cause high FFAs production in the liver [39–41]. High FFAs levels in the liver will then (1) bind and polarize Kupffer cells in the liver into M1, which leads to increased production of proinflammatory cytokines, such as IL-1β, Il-6, and TNF-α, (2) processed in beta-oxidation of mitochondria in the liver, leading to increased ROS production and binding to Kupffer cells along with FFAs, and (3) undergo re-esterification into triglycerides, which are stored in the liver in the form of lipid droplets or combine with liver cholesterol to form lipoproteins. Lipoproteins leave the liver in the form of very-low-density lipoprotein (VLDL) and high-density lipoprotein (HDL) in the bloodstream [42–44]. VLDL released from the liver circulates and changes into intermediate-density lipoprotein (IDL) and low-density lipoprotein (LDL). LDL can be modified and bind to receptors in macrophages, transporting cholesterol (cholesteryl ester/CE) to the cells, and forming foam cells in which play a role in atherosclerosis. LDL remnants are then transported to the liver in the form of cholesterol [45, 46]. Meanwhile, HDL is responsible for removing cholesterol from foam cells in the circulation and arterial walls through reverse cholesterol transport. HDL then transports the cholesterol into the liver via scavenger receptor B-I (SR-BI) [47, 48]. A prolonged high-fat diet results in elevated synthesis and circulation of lipoproteins, including VLDL and LDL, increased conversion of VLDL to FFAs, and heightened absorption of HDL by the liver, leading to dyslipidemia and cholesterolemia [20, 21, 49]. Each of the aforementioned pathways described is interrelated and can lead to non-alcoholic fatty liver disease (NAFLD).

**Fig. 6:**
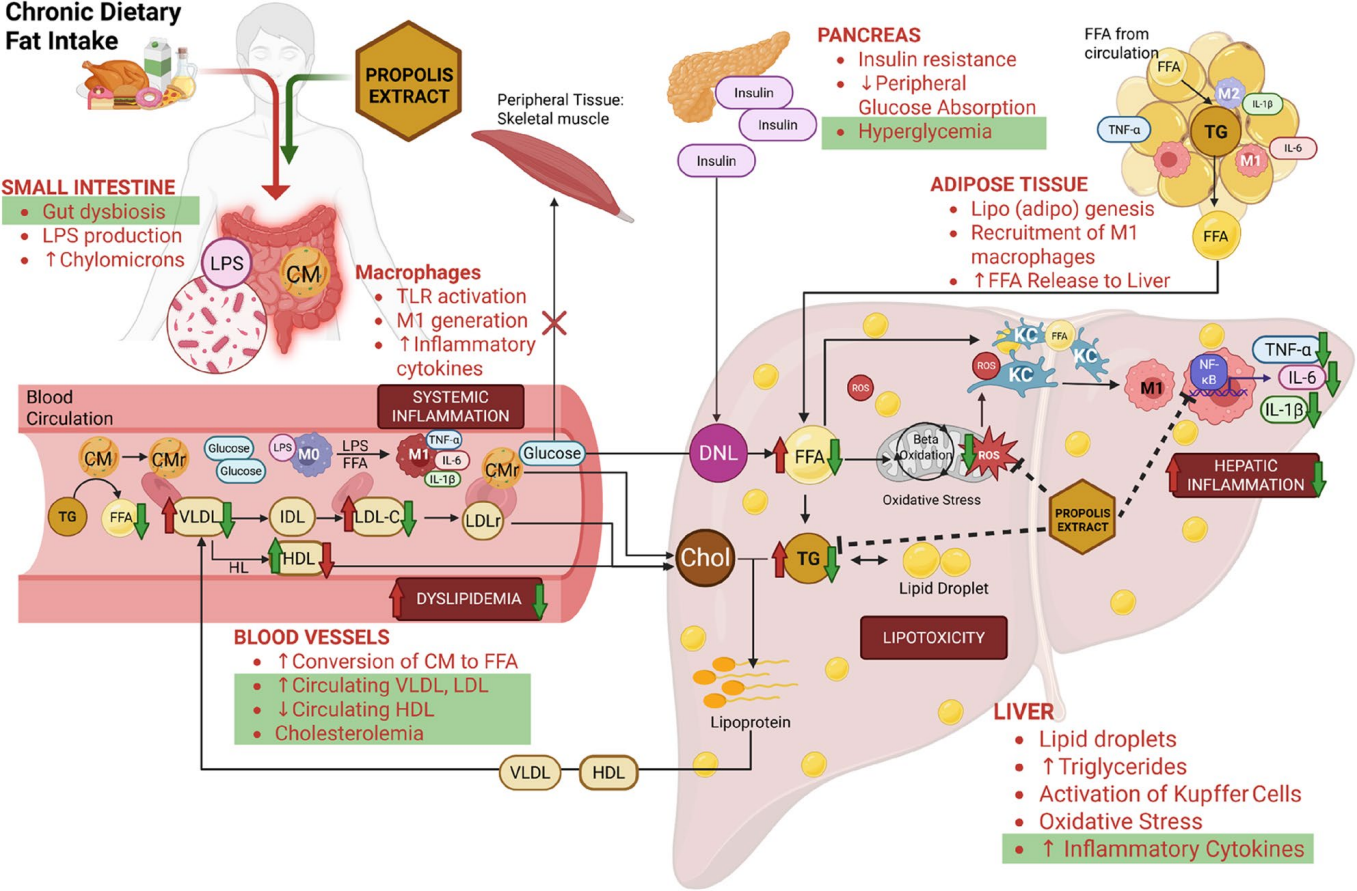
Proposed mechanism by which propolis extract attenuates liver inflammation following a prolonged high-fat diet. The red arrow and red points indicate the effects of a high-fat diet, while the green arrow and shades represent the modulation by propolis extract. LPS: lipopolysaccharides, CM: chylomicrons, CMr: chylomicron remnants, FFA: free fatty acid, TG: triglycerides, TLR: Toll-like Receptor; VLDL: very low-density lipoprotein, IDL: intermediate-density lipoprotein, LDL: low-density lipoprotein, HDL: high-density lipoprotein, DNL: *de novo* lipogenesis, Chol: Cholesterol, ROS: Reactive Oxygen Species

Figure 6 explains the interconnected mechanism of how a high-fat diet affects the liver and circulatory system, as well as the role of propolis extract in mitigating such damage. From our study, we found that Indonesian propolis extract can restore or repair damage caused by a high-fat diet, which we highlighted in Fig. 6, such as restoring gut microbiota to its original state, reducing proinflammatory cytokine production in the liver, such as IL-1β, IL-6, and TNF-α, and lowering circulating cholesterol, triglyceride, LDL, and glucose levels.

Based on these connections, we suggested that propolis might mitigate inflammation by reducing the production of inflammatory cytokines by over 48% through the attenuation of lipid and glucose levels. These relationships revealed that terpenoid-rich GEP can downregulate liver inflammation by lowering dyslipidemia and hyperglycemia. We confirmed that Indonesian propolis has anti-inflammatory and hypolipidemic agents, which help prevent organ damage in metabolic diseases. Similar or subsequent research should examine NF-κB and inflammasome levels because some upstream inflammatory pathways might be involved in HFD-induced inflammation. The other potential of Indonesian propolis in hepatic inflammation can be further investigated in order to impede the development of liver disease. We believe the Indonesian glyceric extract of propolis can repair other organ inflammation in metabolic diseases through several mechanisms.

This study primarily explores the effect of propolis extract on liver inflammation in dyslipidemia after a prolonged high-fat diet; however, we consider two issues that render this study insufficient to represent NAFLD treatment. First, some upstream pathway markers from DNL, Kupffer cells activation, and oxidative stress in liver inflammation are not addressed; and second, the examination of liver histopathology and enzyme markers such as alanine transaminases (ALT) and aspartate transaminases (AST) was not conducted to evaluate the direct effect of propolis extract on liver structure and function. These limitations can be addressed in future research to improve the research on the application of Indonesian propolis extract in the treatment of NAFLD.

## Conclusion

In summary, the Indonesian stingless bees (*Geniotrigona thoracica*) propolis extract could ameliorate hepatic inflammation by improving dyslipidemia and hyperglycemia after chronic HFD administration. Moderate to strong correlation existed between levels of total cholesterol, triglycerides, LDL-C, HDL-C, and glucose, and hepatic inflammatory cytokine expressions of IL-1β, IL-6, and TNF-α. Indonesian propolis demonstrates potential bioactive properties in alleviating metabolic or inflammation-related diseases and disorders.

## Data Availability

The corresponding authors can provide the data supporting the findings of this study upon reasonable request.

## Abbreviations

CD: Control/Normal Chow Diet Group
CP: Control with Propolis Treatment Group
FD: High-Fat Diet Group
FFA: Free Fatty Acid
FP: High-Fat Diet with Propolis Treatment Group
GEP: Glyceric Extract of Propolis
HDL-C: High-Density Lipoprotein-Cholesterol
HFD: High-Fat Diet
IL: Interleukin
JNK: c-Jun N-terminal kinase
LDL-C: Low-Density Lipoprotein-Cholesterol
NAFLD: Non-Alcoholic Fatty Liver Disease
NCD: Normal Chow Diet
NF-κB: Nuclear Factor Kappa-B
ROS: Reactive Oxygen Species
TC: Total Cholesterol
TG: Triglycerides
TNF: Tumor Necrosis Factor
